# Urban Tree Canopy and Asthma, Wheeze, Rhinitis, and Allergic Sensitization to Tree Pollen in a New York City Birth Cohort

**DOI:** 10.1289/ehp.1205513

**Published:** 2013-01-15

**Authors:** Gina S. Lovasi, Jarlath P.M. O’Neil-Dunne, Jacqueline W.T. Lu, Daniel Sheehan, Matthew S. Perzanowski, Sean W. MacFaden, Kristen L. King, Thomas Matte, Rachel L. Miller, Lori A. Hoepner, Frederica P. Perera, Andrew Rundle

**Affiliations:** 1 Department of Epidemiology, Columbia University Mailman School of Public Health, New York, New York, USA; 2 Spatial Analysis Laboratory, University of Vermont, Burlington, Vermont, USA; 3 Division of Forestry, Horticulture and Natural Resources, New York City Department of Parks and Recreation, New York, New York, USA; 4 Institute for Social and Economic Research and Policy, Columbia University, New York, New York, USA; 5 Department of Environmental Health Sciences, Columbia University Mailman School of Public Health, New York, New York, USA; 6 New York City Department of Health and Mental Hygiene, New York, New York, USA

**Keywords:** aeroallergen, allergic sensitivity, asthma, built environment, childhood disease, environmental agents, epidemiology, pollen, urban life

## Abstract

Background: Urban landscape elements, particularly trees, have the potential to affect airflow, air quality, and production of aeroallergens. Several large-scale urban tree planting projects have sought to promote respiratory health, yet evidence linking tree cover to human health is limited.

Objectives: We sought to investigate the association of tree canopy cover with subsequent development of childhood asthma, wheeze, rhinitis, and allergic sensitization.

Methods: Birth cohort study data were linked to detailed geographic information systems data characterizing 2001 tree canopy coverage based on LiDAR (light detection and ranging) and multispectral imagery within 0.25 km of the prenatal address. A total of 549 Dominican or African-American children born in 1998–2006 had outcome data assessed by validated questionnaire or based on IgE antibody response to specific allergens, including a tree pollen mix.

Results: Tree canopy coverage did not significantly predict outcomes at 5 years of age, but was positively associated with asthma and allergic sensitization at 7 years. Adjusted risk ratios (RRs) per standard deviation of tree canopy coverage were 1.17 for asthma (95% CI: 1.02, 1.33), 1.20 for any specific allergic sensitization (95% CI: 1.05, 1.37), and 1.43 for tree pollen allergic sensitization (95% CI: 1.19, 1.72).

Conclusions: Results did not support the hypothesized protective association of urban tree canopy coverage with asthma or allergy-related outcomes. Tree canopy cover near the prenatal address was associated with higher prevalence of allergic sensitization to tree pollen. Information was not available on sensitization to specific tree species or individual pollen exposures, and results may not be generalizable to other populations or geographic areas.

In the United States, a disproportionate burden of severe childhood asthma affects families in poverty, racial or ethnic minorities, and inner-city communities (Akinbami and Schoendorf 2002; Busse and Mitchell 2007; Grant et al. 2000; Matricardi et al. 2002). Recent evidence points to intraurban variability in exposure to air pollution (Carlsten et al. 2011; Padhi and Padhy 2008), allergens (Olmedo et al. 2011), environmental tobacco exposure (Cook and Strachan 1999; Gilliland et al. 2001; Larsson et al. 2001), lifestyles (e.g., sedentary behavior, diet, obesity) (Eder et al. 2006; Platts-Mills et al. 2006; Yang et al. 2007), and aspects of the social environment (Chen et al. 2008; Clougherty et al. 2007; Kilpellainen et al. 2002) that may affect the incidence of asthma or allergic sensitization, a key asthma risk factor (Illi et al. 2006; Porsbjerg et al. 2006; Tuchsen and Hannerz 2000).

Urban landscape elements, particularly trees, have the potential to affect air flow, air quality, and the production of aeroallergens (Bealey et al. 2007; Escobedo et al. 2008; McPherson et al. 1997; Pincus and Stern 1937; Tallis et al. 2011). Air quality and respiratory health improvements are among the anticipated economic and social benefits of the urban forest (Bloomberg 2007; Nowak 2007). Trees may cause small reductions in particulate matter and ozone concentrations (Escobedo et al. 2008; McPherson et al. 1997; Nowak et al. 2000), though evidence directly linking trees to neighborhood air quality is limited. However, one study reported that more vegetation within a 100-m or 250-m buffer around the home was associated with lower personal exposure to particulate matter ≤ 2.5 µm (PM_2.5_) (Dadvand et al. 2012). Some tree species also produce allergens, which may trigger seasonal allergic rhinoconjunctivitis or asthma exacerbations in sensitized individuals (Dales et al. 2004; Larsson et al. 2001; Ridolo et al. 2007). Short-term variation in pollen concentration has been associated with allergy medication purchases (Sheffield et al. 2011), asthma symptoms (DellaValle et al. 2012), and asthma-related emergency department visits (Jariwala et al. 2011; Orazzo et al. 2009).

The role of the urban forest in asthma and allergy prevalence warrants further investigation, especially as governments and organizations in New York City (Bloomberg 2007) and around the world (United Nations Environment Programme 2008) invest substantial resources toward large-scale tree planting efforts. We use data from a birth cohort based in New York City to examine the relationship of total urban tree canopy coverage with childhood asthma, wheeze, rhinitis, and allergic sensitization at the individual level.

## Methods

*Study population: the CCCEH birth cohort in New York City*. The Columbia Center for Children’s Environmental Health (CCCEH) birth cohort (Perera et al. 2002, 2003; Perzanowski et al. 2006) recruited a convenience sample of pregnant women through prenatal clinics. All participants were African American or Dominican and lived in economically disadvantaged areas of New York City (Northern Manhattan and the Bronx). Pregnant women with HIV were excluded. The 727 cohort births occurred during 1998–2006. Children have been followed up through a series of clinical visits for neurodevelopmental, growth, respiratory, and other outcomes. The number of individuals assessed for each outcome depended on loss to follow-up and on the correspondence between data collection efforts for fixed calendar years and ages of the cohort children. Women provided written informed consent at each visit, children also provided assent if they were ≥ 7 years of age, and all study procedures were approved by the Columbia University Medical Center Institutional Review Board.

*Outcome assessment*. Asthma, wheeze, and rhinitis were assessed by parental report collected using the previously validated Brief Respiratory Questionnaire (BRQ) and International Study of Asthma and Allergies in Childhood (ISAAC) questionnaire (Beasley et al. 1998; Bonner et al. 2006). The BRQ asks whether the child has ever been diagnosed by a physician as having asthma. The ISAAC questionnaire asks about wheezing or whistling in the chest during the preceding 12 months, and whether the child has ever had “hay fever,” the lay term used for rhinitis in the ISAAC questionnaire. Questionnaires were completed at approximately 5 years of age (mean ± SD = 60.0 ± 3.7 months) and 7 years (mean± SD = 84.1 2.7 months).

The study visit at 7 years of age included serum IgE antibody testing. A positive IgE test may indicate allergic sensitization of clinical importance (Berg and Johansson 1974; Wide 1973), but may also be present in asymptomatic individuals (Bousquet et al. 2006). Assessment of IgE was conducted with ImmunoCAP® (Phadia, Uppsala, Sweden) for nine specific allergens: German cockroach, mouse urine proteins, dust mites (*Dermatophagoides farinae)*, cat dander, dog dander, mold, common ragweed, mixed grass pollen (Gx2), and mixed tree pollen (Tx1). The mixed tree pollen used for IgE testing included the following species: *Acer negundo* (boxelder), *Betula verrucosa* (European white birch), *Juglans californica* (California black walnut), *Quercus alba* (white oak), and *Ulmus americana* (American elm). Children were classified as sensitized to an allergen based on a specific IgE antibody response ≥ 0.35 IU/mL against the allergen.

*Exposure assessment: urban tree canopy coverage*. A detailed and highly accurate characterization of the urban tree canopy was conducted for the years 2001 and 2010 (MacFaden et al. 2012). The 2010 tree canopy was mapped and used as the basis for estimating the distribution of tree canopy in 2001, which has a better temporal correspondence with the prenatal addresses. The 2010 tree canopy data layer was developed using an automated object-based image analysis approach (O’Neil-Dunne et al. 2009) in eCognition Developer 8.64 software (Trimble, Westminster, CO) that combined high-resolution Light Detection and Ranging (LiDAR) data from an April 2010 flight over New York City, color infrared aerial imagery from spring 2008 (Sanborn Map Company, Colorado Springs, CO), and ancillary vector data from the New York City Department of Parks and Recreation, Department of City Planning, and Department of Information Technology and Telecommunication; New York State Department of Conservation; and U.S. Fish and Wildlife Service; the data derived from these sources are described in detail by MacFaden et al. (2012). The automated approach was followed by a manual review of the data as described previously (MacFaden et al. 2012). Color infrared summer (leaf-on) and true color spring (primarily leaf-off) high-resolution aerial imagery from the year 2001 (New York City Department of Information Technology and Telecommunication, unpublished data) was used as the basis for manually modifying the 2010 tree canopy layer to 2001 conditions, once again at a scale of 1:1,000. There was a high level of agreement using the 2001 or 2010 versions of the tree canopy layer (correlation = 0.98 for 0.25-km buffers around prenatal addresses). Tree canopy coverage was defined as the estimated percent of land area covered by tree canopy.

*Neighborhood definition and characteristics using GIS (geographic information system)*. We focused primarily on prenatal address (reported at the time of recruitment during the third trimester of pregnancy and available for 99.6% of the cohort) to estimate exposures before the onset of the health outcomes. Geocoding was done using Geosupport software, a highly accurate parcel-based address matching program developed by the New York City Department of City Planning. Circular buffers with a radius of 0.25 km were created as the primary neighborhood definition to capture small-scale spatial variation in tree canopy coverage that may be relevant for personal air pollution and aeroallergen exposures. A spatial overlay function within ArcGIS version 10.0 (ESRI, Redlands, CA) was used to intersect these buffers with the tree canopy layer and other geographies used to measure potential confounders: park coverage, traffic volume, and residential composition.

Park coverage was defined as the percent of land area covered by park land based on 2008 data from the New York City Department of Parks and Recreation. Average daily traffic volume was estimated by combining U.S. Census Bureau Feature Class Codes with street network data assembled in 2002 by TeleAtlas from federal, state, county, and city departments (TeleAtlas North America Inc., Lebanon, NH). Census block group data from the 2000 U.S. Census (U.S. Census Bureau 2002) were used to measure neighborhood demographic and socioeconomic characteristics (including percent poverty, defined as the percentage of residents with incomes below the federal poverty line), with block group–level data aggregated to neighborhood buffers using aerial weighting interpolation.

For most in this cohort, the address provided during the third trimester of pregnancy was different from the geocoded address at 5 or 7 years of age; 62% had moved to a new address before their age 5 visit, and 67% had moved to a new address before their age 7 visit. Therefore, alternate neighborhood specifications for sensitivity analyses included estimated tree canopy exposures based on addresses reported at age 5 or 7 years instead of the prenatal address as well as an alternate geographic scale (using larger 1.0-km buffers). Tree canopy coverage and other neighborhood characteristics for larger buffers or in buffers constructed around the age 5 and age 7 addresses were defined using methods parallel to those for the main analysis, except that tree canopy estimates for 2010 (rather than 2001) were used for buffers surrounding age 5 and age 7 addresses.

*Covariates collected via survey and personal monitoring*. During the third trimester of pregnancy, expectant mothers were interviewed to assess sociodemographic characteristics including Medicaid enrollment and ethnicity. Maternal asthma history was assessed by self-report of a physician diagnosis. Environmental tobacco smoke exposure was classified based on the mother’s report of a smoker in the household during pregnancy. Mothers were excluded from the cohort study if they reported active smoking during an initial screening interview. However, mothers who were enrolled and subsequently determined to be active smokers during pregnancy (based on subsequent self-report, medical record information, or cord blood cotinine > 25 ng/mL) were included in the current analyses and classified as active smokers during pregnancy. Maternal ambient airborne polycyclic aromatic hydrocarbon (PAH) exposure during the third trimester was classified as high if the personal air monitor levels were > 2.26 g/m^3^ (Perera et al. 2009).

*Statistical analyses*. Analyses were conducted in Stata 12.0 (StataCorp, College Station, TX), with *p* < 0.05 interpreted as statistical significance. Regression models used robust standard errors that account for clustering of observations within community districts (59 named areas of New York City, governed by community boards). Relative prevalences [referred to as relative risks (RR) for simplicity] were estimated using a log link and Poisson working variance distribution (McNutt et al. 2003). Percent tree canopy coverage in the residential neighborhood was rescaled to have an SD of 1. RRs presented in the tables can be interpreted as the prevalence ratio comparing children whose prenatal neighborhoods differed by 1 SD in tree canopy coverage. Covariates included sex, age at the time of outcome measurement, ethnicity, maternal asthma, previous birth, other previous pregnancy, Medicaid enrollment, tobacco smoke in the home, active maternal smoking, and the following characteristics of 0.25-km buffers: population density, percent poverty, percent park land, and estimated traffic volume.

Covariate data were missing for maternal asthma in 30% of participants, and small numbers (< 1%) were missing data for tobacco smoke in the home and Medicaid enrollment. Although our main analytic strategy was a complete cases analysis (i.e., with observations that had missing data for any covariate excluded), we conducted sensitivity analyses with missing covariate data imputed using multiple imputation (Sterne et al. 2009) or with potential bias due to incomplete follow-up addressed through inverse probability weighting (Hernan et al. 2004). Input variables for both methods included canopy coverage, all neighborhood covariates and individual characteristics included as model covariates, plus high prenatal PAH exposure, which may have predicted loss to follow-up in previous cohort analyses (Rundle et al. 2012). Multiple imputation also used outcome data, and was implemented using chained equations through the *ice* command with parameter estimates combined across 20 imputed data sets using *mi estimate* in Stata. Inverse probability weights were estimated for each outcome using logistic regression models predicting successful follow-up and data collection for age 5 questionnaire data, age 7 questionnaire data, and age 7 IgE testing. Inverse probability weights ranged from 1.1 to 8.3. Models were evaluated using a Hosmer–Lemeshow test and visual inspection across deciles, both of which suggested good fit to the data (data not shown).

## Results

Of 727 participants enrolled, 549 participants had outcome information available based on questionnaire data at 5 (*n* = 492) or 7 years of age (*n* = 427), or based on IgE testing at age 7 (*n* = 288) (Table 1). The prevalence of maternally reported physician-diagnosed asthma was 28% at age 5 and 36% at age 7. Of the children who completed IgE testing, 45% had allergic sensitization to one or more specific allergens. The most common allergic sensitizations were to German cockroach (31%) and mixed tree pollen (19%).

**Table 1:** Child, maternal, household, and neighborhood characteristics.

Characteristic	Enrolled in prenatal sample (n = 727)	Visit completed at age 5 years (n = 492)	Visit completed at age 7 years (n = 427)	IgE antibodies assessed at age 7 years (n = 288)
Child [n (%)]
Male	351 (48)	228 (46)	197 (46)	137 (48)
Dominican	473 (65)	301 (61)	254 (59)	161 (56)
Black	254 (35)	191 (39)	173 (41)	127 (44)
Mother and household [n (%)]
Tobacco smoke in the home during pregnancy	246 (34)	155 (32)	149 (35)	113 (40)
Active maternal smoking during pregnancy	79 (11)	46 (9)	46 (11)	34 (12)
PAH exposurea	347 (51)	224 (48)	210 (51)	159 (57)
Medicaid	657 (91)	450 (92)	388 (91)	266 (92)
Maternal asthma	114 (22)	79 (21)	66 (23)	51 (27)
Mother reports previous live birth	399 (55)	264 (54)	231 (54)	157 (55)
Mother reports any other previous pregnancy (e.g., miscarriage, stillbirth)	149 (21)	108 (22)	94 (22)	69 (24)
Child health outcomes assessed during follow-up [n (%)]
Asthma diagnosed by age 5b		137 (28)
Wheeze at age 5c		126 (26)
Rhinitis at age 5c		15 (3)
Asthma diagnosed by age 7b			152 (36)
Wheeze at age 7c			106 (25)
Rhinitis at age 7c			20 (5)
Any specific allergic sensitizationd				131 (45)
Allergic sensitization to tree pollend				54 (19)
0.25-km buffer prenatal neighborhood (mean ± SD)
Population density (thousands/km2)	43 ± 14	42 ±14	43 ± 14	42 ± 14
Area poverty (percent of residents below federal poverty line)	37 ± 7	37 ± 7	37 ± 7	37 ± 7
Area racial composition (percent of residents reporting black race)	40 ± 27	42 ± 28	43 ± 28	45 ± 28
Traffic volume (estimated average daily traffic in thousands of vehicles)	11 ± 4	11 ± 4	11 ± 4	11 ± 4
Park coverage (percent of land area covered by park land)	8 ± 9	8 ± 9	8 ± 9	8 ± 9
Tree canopy coverage (percent of land area covered by tree canopy)	15 ± 8	16 ± 8	16 ± 8	16 ± 8
a Polycyclic aromatic hydrocarbon (PAH) exposure was dichotomized based on a median split (prenatal personal monitor value of 2.26 g/m3) among participants followed to age 5. bAssessment was based on the BRQ. cAssessment was based on the ISAAC Questionnaire. dIgE antibodies were dichotomized at 0.35 IU/mL to define allergic sensitization to each specific allergen.				

**Figure 1:**
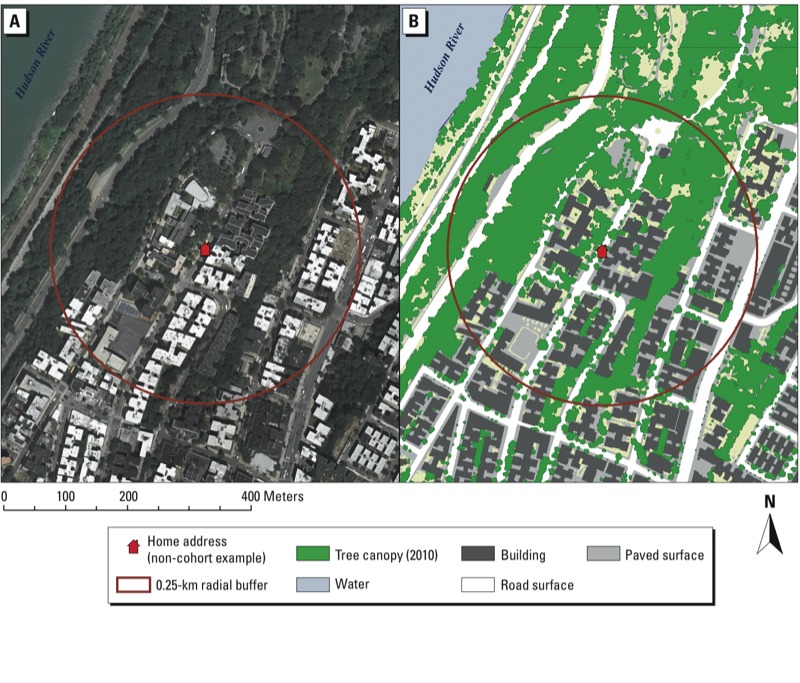
Circular buffer with tree canopy coverage shown using (*A*) orthophotography and (*B*) land use classification. The figure shows an example address within the study area (though for confidentiality reasons the address of a study participant was not used), surrounded by a 0.25-km radial buffer, to illustrate that tree canopy coverage was calculated as the percentage of land area within the circle classified as tree canopy. Data sources are described by MacFaden et al. (2012).

The 0.25-km neighborhood buffers around the prenatal addresses (Figure 1) were characterized by 2–41% canopy coverage, with a mean ± SD of 15 ± 8%. Tree canopy coverage was negatively correlated with population density, and positively correlated with percent poverty, percent black race, daily traffic volume, and percent of area covered by park land (Table 2).

**Table 2:** Neighborhood characteristic correlation matrix.

Neighborhood characteristic	Population density	Area poverty	Area racial composition	Traffic volume	Park coverage
Population density (thousands/km2)
Area poverty (percent of residents below federal poverty line)	–0.32*	—
Area racial composition (percent of residents reporting black race)	–0.55*	0.31*	—
Traffic volume (estimated average daily traffic in thousands of vehicles)	–0.15*	–0.18*	0.09*	—
Park coverage (percent of land area covered by park land)	–0.28*	0.04	–0.04	0.13*	—
Tree canopy coverage (percent of land area covered by tree canopy)	–0.42*	0.14*	0.25*	0.14*	0.59*
Neighborhood characteristics shown were assessed for a 0.25‑km buffer around the prenatal address for 549 participants with one or more outcomes assessed. *p < 0.05.					

**Figure 2:**
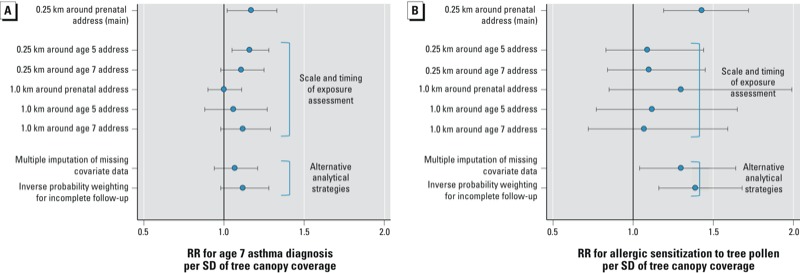
Sensitivity analyses to examine the robustness of associations tree canopy coverage with asthma and allergic sensitization to tree pollen. Values shown are 95% CI and risk ratio (RR) for an association between tree canopy coverage and either (*A*) parental report of physician-diagnosed asthma at 7 years of age or (*B*) allergic sensitization to tree pollen based on IgE testing from sensitivity analysis models adjusting for the following covariates: sex, age at the time of outcome measurement, ethnicity, maternal asthma, previous birth, other previous pregnancy, Medicaid enrollment, tobacco smoke in the home, active maternal smoking, and the following characteristics of 0.25-km buffers: population density, percent poverty, percent park land, and estimated traffic volume.

Proportions of children with asthma, wheeze, or rhinitis did not appear to be related to quartiles of tree canopy coverage near the prenatal address (Table 3). Regression models adjusted for covariates indicated a significant positive association of tree canopy coverage with diagnosed asthma at 7 years of age (adjusted RR = 1.17; 95% CI: 1.02, 1.33) consistent with a 17% increase in the prevalence of asthma with each SD increase in tree canopy coverage (for tree canopy coverage, the SD was 8%). The association was similar but nonsignificant based on models employing either multiple imputation of missing data or inverse probability weighting to adjust for loss to follow-up (Figure 2A). Associations of tree canopy coverage with asthma at 5 years, and with wheeze at 5 or 7 years, were similar in magnitude, but not statistically significant [Table 4; see also Supplemental Material, Figure S1 (http://dx.doi.org/10.1289/ehp.1205513)]. RRs for associations with rhinitis were larger, but were imprecise and not significant due to the low prevalence of this outcome.

**Table 3:** Outcome distributions according to quartiles (Q) of tree canopy coverage (%).^*a*^

	Q1	Q2	Q3	Q4
	2.5–9.5%	9.5–13.5%	13.5–21.1%	21.1–41.4%
Reported asthma diagnosis at age 5 yearsb	26	30	24	31
Wheeze at age 5 yearsc	25	31	23	24
Rhinitis at age 5 yearsc	2	3	4	3
Reported asthma diagnosis at age 7 yearsb	32	36	34	39
Wheeze at age 7 yearsc	25	25	23	26
Rhinitis at age 7 yearsc	2	6	9	2
Any specific allergic sensitizationd	45	48	42	46
Allergic sensitization to tree pollend	12	18	20	26
aQuartiles were created for tree canopy coverage within a 0.25‑km buffer around the prenatal address for 549 participants with one or more of these outcomes assessed. bAssessment was based on the BRQ. cAssessment was based on the ISAAC Questionnaire. dIgE antibodies were dichotomized at 0.35 IU/mL to define allergic sensitization to each specific allergen.				

**Table 4:** Associations per SD of tree canopy coverage near prenatal address with asthma, wheeze, rhinitis, and allergic sensitization.

	Unadjusted RR (95% CI)		p-Value	Adjusteda RR (95% CI)		p-Value
Reported asthma diagnosis at age 5 years	1.06	(0.93, 1.22)	0.39	1.11	(0.85, 1.45)	0.42
Reported asthma diagnosis at age 7 years	1.07	(0.99, 1.16)	0.09	1.17	(1.02, 1.33)	0.02
Wheeze at age 5 years	0.95	(0.82, 1.08)	0.42	1.15	(0.95, 1.39)	0.16
Wheeze at age 7 years	0.98	(0.90, 1.08)	0.75	1.13	(0.97, 1.32)	0.11
Rhinitis at age 5 years	1.14	(0.87, 1.50)	0.36	1.60	(0.79, 3.22)	0.19
Rhinitis at age 7 years	0.89	(0.68, 1.17)	0.40	1.40	(0.63, 3.08)	0.41
Any specific allergic sensitization	1.03	(0.89, 1.19)	0.735	1.20	(1.05, 1.37)	0.008
Allergic sensitization to tree pollen	1.33	(1.16, 1.52)	< 0.001	1.43	(1.19, 1.72)	< 0.001
Values shown are risk ratios for regression models examining the association of local tree canopy cover using 2001 source data for 0.25-km buffers around prenatal address; tree canopy has been rescaled to a z-score so that the RRs shown are for a 1‑SD increase in tree canopy coverage; the cluster robust standard errors for these analyses account for clustering within community districts. aAdjusted models included the following covariates: sex, age at the time of outcome measurement, ethnicity, maternal asthma, previous birth, other previous pregnancy, Medicaid enrollment, tobacco smoke in the home, active maternal smoking, and the following characteristics of 0.25‑km buffers: population density, percent poverty, percent park land, and estimated traffic volume.						

The proportion of children with allergic sensitization to tree pollen increased with increasing quartiles of tree canopy coverage (Table 3, *p* = 0.03). In addition, each 1-SD increase in tree canopy coverage was associated with a 43% increase in the probability of IgE antibody response to the tree pollen mix (95% CI: 1.19, 1.72) after adjustment (unadjusted RR = 1.33; 95% CI: 1.16, 1.52) (Table 4). The association was similar following multiple imputation or inverse probability weighting, but was weaker and no longer significant when based on tree canopy distributions for addresses at ages 5 or 7 years, and when based on a larger 1-km buffer (Figure 2B). Tree canopy coverage also was positively associated with the IgE response to any of the nine allergens after (but not before) adjustment for covariates (RR = 1.20; 95% CI: 1.05, 1.37) (Table 4) and with the IgE response to several individual allergens when modeled separately [see Supplemental Material, Table S1 (http://dx.doi.org/10.1289/ehp.1205513)].

## Discussion

Results from this birth cohort study did not support the hypothesized protective association of urban tree canopy coverage with asthma or wheeze. However, tree canopy cover within 0.25 km of the prenatal address was associated with higher prevalence of allergic sensitization to tree pollen at 7 years of age that was evident before and after covariate adjustment and when analyzed using alternate methods to address missing data and loss to follow-up. Allergic sensitization to tree pollen and other allergens as defined in this study does not necessarily indicate the presence of noticeable allergy symptoms such as rhinitis, which was relatively uncommon in this cohort and was not significantly associated with exposure.

A previously observed inverse association between street trees and asthma (Lovasi et al. 2008) was limited by an ecologic design, with the neighborhood unit of analysis being much larger than the 0.25-km buffer areas evaluated in this study, and did not consider the full geographic extent of urban trees, due to a focus exclusively on street trees. Most trees in New York City’s urban forest would not be included as street trees because they are in public parks or on private property (Nowak 2007). We hypothesized that urban tree canopy coverage would have a protective effect on asthma through a reduction in local air pollution exposure, but we did not observe any trends in the hypothesized direction, and some of our analyses indicated a positive association with the prevalence of asthma among children in our study population. The amount of pollution removed by the urban tree canopy (McPherson et al. 1997; Nowak 2007; Nowak et al. 2000) may be too small to cause meaningful differences in air pollution exposure among New York City residents, particularly given spatial exposure gradients attributable to emissions from vehicles and other urban sources (Gromke 2011).

Urban tree canopy coverage near the prenatal address was associated with IgE response to a tree pollen mix and other allergens (notably grass pollen, ragweed, and cat dander) at 7 years of age in this birth cohort study. Our assessment of tree canopy coverage and allergic sensitization did not allow us to determine the specificity of the association at the level of tree species or to evaluate the role of plant biodiversity (Hanski et al. 2012), and we did not have data on personal or outdoor exposure to tree pollen, which has been previously evaluated alongside tree pollen sensitization as a predictor of rhinitis (Codispoti et al. 2010). The observed associations may be the result of multiple allergen exposures, or other geographically patterned exposures, that are higher in areas of New York City with more tree canopy coverage. However, the tree pollen mix used for the IgE assessment included three species (*Acer negundo, Quercus alba,* and *Ulmus americana*) that each make up at least 1% of the leaf area in New York City (Nowak 2007). In addition, there are cross-reactivities between the five species represented in the tree pollen mix and other tree species of the same genera (White and Bernstein 2003).

Randomized controlled studies of interventions to reduce multiple allergen exposures have suggested that early allergen exposures contribute to later development of allergic sensitization (Arshad et al. 1992; Halmerbauer et al. 2003). Although geographic (Chew et al. 2003; Cohn et al. 2004) and temporal (Asero 2002; Ridolo et al. 2007) variation in early-life allergen exposure also occurs without clinical manipulation, there is limited evidence as to whether such variation predicts allergic sensitization. Tree pollen counts have been reported to be higher in the homes of children with allergic sensitization to tree pollen (Matsui et al. 2010; Warner et al. 1990), but the interpretation of such findings remains unclear; in fact, one recent study suggests that quantitative IgE levels can be used as a biomarker for exposure to indoor allergens among sensitized individuals (Matsui et al. 2010). It is unknown whether individuals without symptomatic allergic sensitization would likewise have higher IgE response and be more likely to exhibit false positive tests for allergic sensitization following allergen exposure, but if so an association between allergen exposure and seroatopy could be observed without having meaningful clinical consequences. Several studies of seasonal exposure patterns suggest an association between pollen exposure and sensitization that may be strongest in infancy rather than during pregnancy (Bjorksten et al. 1980; Kihlstrom et al. 2002; Knudsen et al. 2007; Lendor et al. 2008), but an association between novel allergen exposure and allergic sensitization may also persist into adulthood (Asero 2004). Tree canopy exposure estimates based on the prenatal neighborhood in our study may be serving as a proxy marker of exposure during infancy or early childhood.

Key strengths of this study include detailed assessment of the urban tree canopy using LiDAR and multispectral image processing to characterize buffer neighborhoods centered on prenatal home addresses. Furthermore, we used previously validated outcomes with relevance to asthma or allergic sensitization. In addition, the birth cohort design made it possible to control for detailed, prospectively collected family, home, and neighborhood characteristics that may confound the associations of interest.

Our study was limited by the lack of individual measures of pollen exposure and measures of exposure to specific allergenic tree species. Estimates may be biased by unmeasured or residual confounding, or by cohort selection or attrition. In addition, the correlations among neighborhood characteristics, and particularly between tree canopy and park coverage, limits our ability to distinguish the importance of particular types of green space or vegetation. Measurement error may be present in the health outcomes or the tree canopy exposure metric. In particular, the accuracy of geographic exposure measures could be improved by incorporating other frequently visited locations (Lovasi et al. 2012b). Finally, restriction to a population of African-American and Dominican families in low-income areas of New York City may limit the generalizability of our findings to other populations and geographic areas.

## Conclusions

Although asthma and allergy in children are important urban health concerns, our findings should be considered in the context of considerable environmental benefits of urban tree cover such as carbon sequestration, heat island reduction, energy conservation, and storm water management (McPherson et al. 1997; Nowak 2007) as well as potential health benefits suggested by other studies (Donovan et al. 2011; Lovasi et al. 2011, 2012a; Mitchell and Popham 2008). Further study is needed to elucidate the influence, if any, of the urban forest on allergic and respiratory illness. Future research should examine spatial variation in tree species, pollen exposure, and air quality and their link to health across diverse populations and geographic settings.

## Correction

The original manuscript published online reported that testing used Tx8 instead of the correct Tx1, which included *Juglans californica* (California black walnut) and *Ulmus americana* (American elm) rather than *Corylus avellana* (common filbert) and *Platanus x acerifolia* (London planetree). The text has been corrected here.

## Supplemental Material

(201 KB) PDF
