# Supplementary material for: Urban Tree Canopy and Asthma, Wheeze, Rhinitis, and Allergic Sensitization to Tree Pollen in a New York City Birth Cohort
Source: Environ Health Perspect. 2013 Jan 15;121(4):494–500. doi: 10.1289/ehp.1205513 (PMC3620770; doi:10.1289/ehp.1205513)
Supplement: (201 KB) PDF [file ehp.1205513.s001.pdf]

Supplemental Material

Urban Tree Canopy and Asthma, Wheeze, Rhinitis, and Allergic Sensitization to Tree Pollen in a New York City Birth Cohort

Authors and Affiliations

Gina S. Lovasi,[1] Jarlath P. M. O'Neil-Dunne,[2] Jacqueline W. T. Lu, [3] Daniel Sheehan,[1,4] Matthew S. Perzanowski,[5] Sean W. MacFaden,[2] Kristen L. King,[3] Thomas Matte,[6] Rachel L. Miller,[5] Lori A. Hoepner,[5] Frederica P. Perera,[5] Andrew Rundle[1]

[1]Columbia University Mailman School of Public Health, Department of Epidemiology, New York City, New York, USA

[2]University of Vermont, Spatial Analysis Laboratory, Burlington, Vermont, USA

[3]New York City Department of Parks & Recreation, Division of Forestry, Horticulture & Natural Resources, New York City, New York, USA

[4]Columbia University, Institute for Social and Economic Research and Policy, New York City, New York, USA

[5]Columbia University Mailman School of Public Health, Department of Environmental Health Sciences, New York City, New York, USA

[6]New York City Department of Health and Mental Hygiene, New York City, New York, USA

Table of Contents

Supplemental Material, Figure S1. Sensitivity Analyses to Examine the Robustness of Associations Tree Canopy Coverage with Asthma, Wheeze, and Allergic Sensitization (Described on Page 2, shown on Page 3)

Supplemental Material, Table S1. Relative risks for associations of tree canopy coverage near prenatal address with IgE test results for allergic sensitization at age 7 years (Described on Page 4, shown on Pages 5-6)

This supplemental material is designed to highlight sensitivity analyses to complement the tables and figures presented in our manuscript.

Sensitivity analyses are presented for two outcomes in Figure 1 of the manuscript, and an additional 4 panels are shown here (Figure S1). Rhinitis has been omitted due to limited statistical power for this outcome. In each panel, a reference line is provided at a relative risk of 1. All 95% confidence intervals either cross this line, indicating a lack of statistical significance, or are to the right of the line, indicating an association of tree canopy coverage with increased risk. We do not observe any significant association of tree canopy coverage with lower risk of asthma, wheeze, or allergic sensitization.

Figure S1. Sensitivity analyses to examine the robustness of associations tree canopy coverage with asthma, wheeze, and allergic sensitization

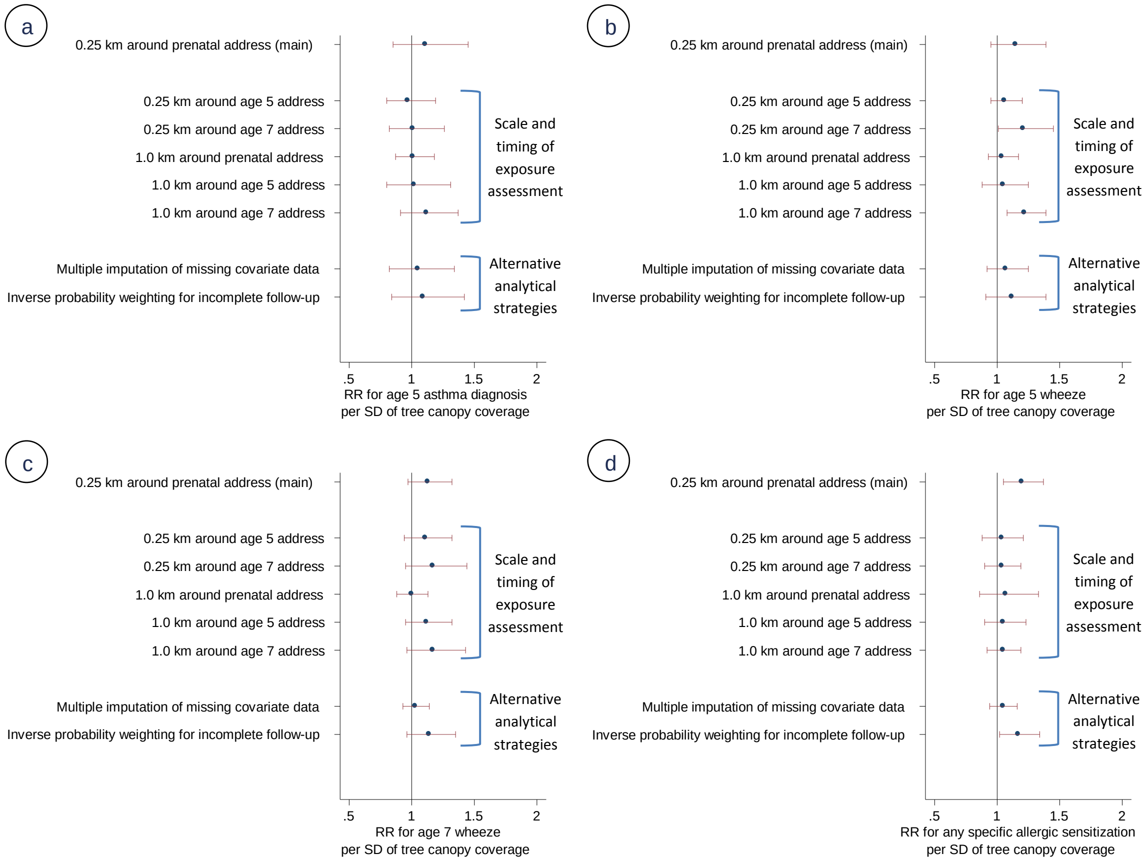

Notes: The figure shows relative risks and 95% confidence intervals for an association between tree canopy coverage and parental report of physician diagnosed asthma (a), wheeze (b, c) or allergic sensitization based on IgE testing (d) from sensitivity analysis models adjusting for the following covariates: sex, age at the time of outcome measurement, ethnicity, maternal asthma, previous birth, other previous pregnancy, Medicaid enrollment, tobacco smoke in the home, active maternal smoking, and the following characteristics of 0.25 km buffers: population density, percent poverty, percent park land, and estimated traffic volume. A vertical line has been drawn at an RR equal to one for reference; estimates to the right of this line indicate tree canopy coverage was associated with a higher risk; 95% confidence intervals that do not cross this line represent statistically significant associations.

There were several significant associations suggesting that tree canopy coverage was associated with allergic sensitization not only to tree pollen, but also to cat and dog dander, mold, ragweed, and perhaps to grass pollen and German cockroach (Table S1). Allergic sensitization to any one of the specific allergens significantly predicts allergic sensitization to tree pollen ($p<0.001$).

Table S1. Associations of tree canopy coverage near prenatal address with IgE test results for allergic sensitization at age 7 years

Childhood health outcome	Associations per SD tree canopy coverage Unadjusted RR (95% CI)	p-value	Adjusted* RR (95% CI)	p-value
**Allergic sensitization to**				
**German cockroach**	1.13 (0.99 to 1.28)	0.071	1.35 (1.16 to 1.57)	<0.001
**Mouse urine proteins**	1.04 (0.83 to 1.30)	0.744	1.30 (0.97 to 1.75)	0.081
**Dust mites (*D. farina)***	1.12 (0.95 to 1.32)	0.164	1.22 (0.82 to 1.82)	0.326
**Cat dander**	1.25 (1.13 to 1.39)	<0.001	2.10 (1.61 to 2.72)	<0.001
**Dog dander**	1.28 (1.00 to 1.63)	0.049	1.62 (1.13 to 2.34)	0.009
**Mold**	1.71 (1.21 to 2.42)	0.002	2.91 (1.81 to 4.70)	<0.001
**Common ragweed**	1.32 (1.01 to 1.73)	0.04	1.79 (1.47 to 2.18)	<0.001
**Grass pollen**	1.38 (0.94 to 2.03)	0.103	2.30 (1.73 to 3.06)	<0.001
**Tree Pollen**	1.33 (1.16 to 1.52)	<0.001	1.43 (1.19 to 1.72)	<0.001

Note: Values shown are risk ratios for regression models examining the association of local tree canopy cover using 2001 source data for 0.025 km buffers around prenatal address; tree canopy has been rescaled to a z-score so that the RRs shown are for a 1 standard deviation increase in tree canopy coverage; these generalized estimating equation (GEE) analyses account for clustering within community districts

*Adjusted models included the following covariates: sex, age at the time of outcome measurement, ethnicity, maternal asthma, previous birth, other previous pregnancy, Medicaid enrollment, tobacco smoke in the home, active maternal smoking, and the following characteristics of 0.25 km buffers: population density, percent poverty, percent park land, and estimated traffic volume
